# Design and Characterization of Phosphatidylcholine-Based Solid Dispersions of Aprepitant for Enhanced Solubility and Dissolution

**DOI:** 10.3390/pharmaceutics12050407

**Published:** 2020-04-29

**Authors:** Sooho Yeo, Jieun An, Changhee Park, Dohyun Kim, Jaehwi Lee

**Affiliations:** College of Pharmacy, Chung-Ang University, Seoul 06974, Korea; sooho32@cau.ac.kr (S.Y.); missfrog@cau.ac.kr (J.A.); goodchangepark@cau.ac.kr (C.P.); dylan007@cau.ac.kr (D.K.)

**Keywords:** aprepitant, phospholipid, solid dispersion, solubility, dissolution, antiemetic

## Abstract

This study aimed to improve the solubility and dissolution of aprepitant, a drug with poor aqueous solubility, using a phosphatidylcholine (PC)-based solid dispersion system. When fabricating the PC-based solid dispersion, we employed mesoporous microparticles, as an adsorbent, and disintegrants to improve the sticky nature of PC and dissolution of aprepitant, respectively. The solid dispersions were prepared by a solvent evaporation technique and characterized by Fourier transform infrared spectroscopy (FTIR), differential scanning calorimetry, and X-ray powder diffraction. The FTIR results showed that aprepitant interacted with the PC carrier by both hydrogen bonds and van der Waals forces that can also be observed in the interaction between aprepitant and polymer carriers. The solid dispersions fabricated with only PC were not sufficient to convert the crystallinity of aprepitant to an amorphous state, whereas the formulations that included adsorbent and disintegrant successfully changed that of aprepitant to an amorphous state. Both the solubility and dissolution of aprepitant were considerably enhanced in the PC-based solid dispersions containing adsorbent and disintegrant compared with those of pure aprepitant and polymer-based solid dispersions. Therefore, these results suggest that our PC-based solid dispersion system is a promising alternative to conventional formulations for poorly water-soluble drugs, such as aprepitant.

## 1. Introduction

Aprepitant (APR) is one of the drugs approved for the treatment of acute and delayed nausea and vomiting in cancer patients undergoing chemotherapy [1,2,3]. Currently, APR is available in capsule formulation of different doses (40, 80, and 125 mg) marketed by Merck and Company, Inc.(Kenilworth, NJ, USA) [4]. The molecular weight of APR is 534 g/mol, and its chemical name is 3-{[(2R,3S)-2-[(1R)-1-[3,5-bis(trifluoromethyl)phenyl]ethoxy]-3-(4-fluorophenyl)morpholin-4-yl]methyl}-4,5-dihydro-1H-1,2,4-triazol-5-one.

APR is a relatively highly lipophilic compound, as it exhibits a log P value of 4.8 at pH 7.0 [5]. APR suffers considerably from formulation challenges since it has very low aqueous solubility, reported to be 3–7 μg/mL over the pH range of 2–10 [6,7,8,9]. Thus, the oral bioavailability of APR is known to be limited by its solubility and dissolution process [4,7,10,11]. The dissolution process of poorly soluble drugs is usually the rate-limiting step for their gastrointestinal absorption [12]. Therefore, it would be advantageous to increase the solubility and dissolution rate of APR to develop efficient dosage forms.

Various approaches have been proposed for improving the solubility and dissolution of poorly soluble drugs, such as nanonization [5,8,9], amorphization [13,14], inclusion complexation with cyclodextrin [4,10,15], self-micro/nano emulsifying drug delivery systems [16,17,18], and solid dispersion systems [19,20]. Specifically, Merck and Company, Inc. has developed a commercial formulation of APR called Emend, which is based on nanoparticle technology to enhance the solubility and dissolution rate of APR; this formulation exhibits an oral drug bioavailability of ~65% (Merck and Company, Inc.) [4,6,10,11]. However, considering that nanonization is a time-consuming process and requires investment in specialized facilities, pharmaceutical technologies for enhancing the solubility and dissolution rate of APR that are alternatives to this complicated technology should be investigated [8,21].

Solid dispersion systems appear to be a simple and very efficient approach for increasing the aqueous solubility and dissolution rates of poorly water-soluble drugs [6,10]. In solid dispersion systems, a hydrophobic, poorly soluble drug is molecularly dispersed in a hydrophilic polymeric matrix to alter the crystalline state of the drug to an amorphous state, which effectively improves its solubility and dissolution rate [22,23,24]. The success of the development of efficient solid dispersion systems depends significantly on the selection of carriers [14]. In this regard, many investigators are still searching for ideal carrier materials to be used for the molecular dispersion of poorly soluble drugs [25]. Various hydrophilic polymers, such as polyethylene glycols (PEGs), polyvinyl pyrrolidone (PVP), hydroxypropyl methylcellulose (HPMC), and poloxamers, have been frequently used to prepare solid dispersion systems as carrier materials for this purpose [26,27,28]. However, polymer-based solid dispersion systems are known to absorb atmospheric moisture, which is a concern for storage stability as well as the possibility of phase-separation and conversion of the amorphous state to the crystalline form. This may also decrease the solubility and dissolution rate of the drug over time [29,30]. Therefore, the limitations of polymer-based carriers may need to be overcome.

A literature search has revealed that lipid-based systems are promising candidates to improve the bioavailability of poorly soluble drugs [1,31,32]. When considering the lipophilic nature of APR, the molecular dispersion of APR in lipids should be straightforward [2,3,5], and phospholipids are the most promising in terms of safety, cost, and biocompatibility [11,33]. The amphiphilic property of phospholipids could facilitate the entrapment and dispersion of poorly soluble drugs [34,35]. However, phospholipids are known to be rather sticky and difficult to formulate as solid dosage forms, which would pose a challenge when compared to polymer-based solid dispersion systems [36,37]. Therefore, lipid-based dispersion systems of poorly soluble drugs need to be transformed to a solid state for better handling and processability [38,39].

In the present work, phospholipid-based solid dispersions of APR were prepared to improve its solubility and dissolution rates in the solid state. To impart solid state properties to the APR phospholipid-based dispersion system, inorganic mesoporous excipients were employed to adsorb the lipid-based dispersions and thereby improve their powder properties. As the adsorption of APR in the phospholipid-based dispersion system resulted in delayed APR release, promoting the release of the drug was also an aim of this study.

## 2. Materials and Methods

### 2.1. Materials

APR, croscarmellose sodium (CCS), Kollidon^®^ CL (crospovidone), polyvinylpyrrolidone K30 (PVP K30), and polyethylene glycol 6000 (PEG 6000) were supplied by BASF (Ludwigshafen, Germany). Neusilin^®^ US2 (magnesium aluminometasilicate) was a generous gift from Wooshin Labottach, Ltd. (Seoul, Korea). Syloid^®^ 244 FP Silica (silicone dioxide) was purchased from Grace Co., Ltd. (Columbia, MD, USA). Phosphatidylcholine (PC) was obtained as a generous gift from Lipoid (Ludwigshafen, Germany). Distilled and deionized water was purchased from Dae Jung Co., Ltd. (Busan, Korea). All other chemicals utilized were of reagent or pharmaceutical grade.

### 2.2. Preparation of PC-Based Solid Dispersions

PC-based dispersions of APR were first prepared by a solvent evaporation method. Briefly, APR and a predetermined amount of PC, as shown in Table 1, were dissolved in ethanol with/without adsorbents (Neusilin^®^ or Syloid^®^) and disintegrants (CCS or Kollidon^®^ CL). The disintegrants were employed to promote water uptake leading to prompt separation of the drug from the adsorbents. With gentle stirring for 1 h, ethanol was removed using a vacuum rotary evaporator (Vacuum Rotavaporator, R-210, Büchi Corp., New Castle, DE, USA). The obtained dispersions were dried in an oven at 50 °C for 12 h. The dried dispersions were then passed through a 140 mesh (USA standard sieve) with particle sizes around 100–110 μm. The composition ratio of APR and adsorbents was 1:2, while that of APR and disintegrants was 2:1.

Polymer-based solid dispersions as traditional solid dispersion systems were also prepared to compare their dispersion characteristics with those of the PC-based dispersion formulations. These polymer-based solid APR dispersions were prepared by the same technique under the same conditions. The composition ratio of APR to polymers (PVP K30 or PEG 6000) was 1:3.

### 2.3. Instrument Characterization of Solid Dispersions

#### 2.3.1. Fourier Transform Infrared Spectroscopy (FTIR)

To obtain direct information about the chemical interaction between APR and the solid dispersion ingredients, such as PC, Neusilin^®^, PVP K30, and PEG 6000, FTIR was carried out using an FTIR spectrometer (TENSORII, Bruker, Germany). The samples were mixed thoroughly with potassium bromide (KBr) at 1:100 (sample:potassium bromide) weight ratio. After being pulverized, the powders were compressed under high pressure to prepare the potassium bromide pellets. The spectra were recorded over the wavenumber range of 4000 to 440 cm^−1^.

#### 2.3.2. Differential Scanning Calorimetry (DSC)

DSC was used to study the thermodynamic properties of pure APR, pure PC, pure Neusilin^®^, the solid dispersions, and their corresponding physical mixtures. DSC curves were obtained with a Differential Scanning Calorimeter (STA S-1000, Scinco, Seoul, Korea). An appropriate amount of each sample was loaded into an aluminum sample pan for each run. The thermal cycle was performed using a 10 °C/min heating rate from 30 to 350 °C.

#### 2.3.3. Morphological Evaluation

Morphological evaluation was performed to compare the morphologies and particle sizes of pure APR, PC-based formulations with/without adsorbents and/or disintegrants, and polymer-based formulations. Ten milligrams of each sample was gently mounted onto a glass plate. The morphologies of the solid dispersions were then examined by microscopy (Axio Imager.A1m, Zeiss Co., Oberkochen, Germany) using crossed polarizers with an Epi DF reflector, a 10× eyepiece lens, and a 20× objective lens.

#### 2.3.4. X-ray Powder Diffraction (XRPD)

The XRPD patterns of APR and the ingredients for preparing the solid dispersion were studied using an X-ray diffractometer (D8 Advance, Bruker AXS, Germany). The appropriate amount of each sample was put into the plate. X-ray diffraction patterns were obtained by using Cu-Ka radiation (1.54056 Å) at 40 kV, 100 mÅ, 0.3° divergence and anti-scatter slits, 3 degree/min scan speed, and a range of 3–40°.

### 2.4. Evaluation of Physical Properties of Powderized Solid Dispersions

#### 2.4.1. Measurement of Powder Density

Bulk density was determined by pouring pre-weighed and pre-sieved dispersion formulations into a graduated cylinder via a large funnel, and the volume was measured and recorded as the bulk volume. The cylinder was tapped until the powder bed volume reached a minimum, and this volume was recorded as the tapped volume. The bulk density and tapped density were calculated from Equations (1) and (2), respectively.

(1) $$\mathrm{Bulk}\;\mathrm{density}\;\left(g/\mathrm{mL}\right)=\frac{\mathrm{Weight}\;\mathrm{of}\;\mathrm{sample}}{\mathrm{Bulk}\;\mathrm{volume}}$$

(2) $$\mathrm{Tapped}\;\mathrm{density}\;\left(g/\mathrm{mL}\right)=\frac{\mathrm{Weight}\;\mathrm{of}\;\mathrm{sample}}{\mathrm{Tapped}\;\mathrm{volume}}$$

#### 2.4.2. Measurement of Compressibility

Carr’s index is known as percent compressibility, indirectly related to the flow rate, cohesiveness, and particle size. Compressibility is the ability of a powder to decrease in volume under pressure. Carr’s compressibility index of the powder was obtained from density determinations. It is a simple, fast, and accurate method of predicting powder flow characteristics. Carr’s index for each formulation was calculated using Equation (3) [40,41].

Hausner ratio, the ratio of tapped density to bulk density, is related to interparticle friction and can be used to predict powder flow properties. The Hausner ratio for each formulation was calculated using Equation (4) [40,41].

(3) $$\mathrm{Carr}’s\;\mathrm{Index}\;\left(\%\right)=\frac{\mathrm{Tapped}\;\mathrm{density}\;-\;\mathrm{Bulk}\;\mathrm{density}}{\mathrm{Tapped}\;\mathrm{density}}\times 100$$

(4) $$\mathrm{Hausner}\;\mathrm{ratio}=\frac{\mathrm{Tapped}\;\mathrm{density}}{\mathrm{Bulk}\;\mathrm{density}}$$

#### 2.4.3. Measurement of Flow Properties

The flowability of solid dispersions was evaluated by measuring the angle of repose and the flow rate through a funnel. For measurement of the angle of repose, a fixed funnel method was used. Specifically, a fixed funnel (Copley Scientific Ltd., Nottingham, UK) with an orifice inner diameter of 10 mm and specially designed to evaluate the flowability of powders or granules was used. The funnel was fixed above a flat horizontal surface at an appropriate height. The orifice of the funnel was closed, and 10 g of solid dispersion was poured into the funnel. Then, the orifice of the funnel was opened, allowing the solid dispersion to pass through. The angle of repose (θ) of the conical piles formed by each formulation was calculated using Equation (5), after measuring the height (h) of each conical pile and the radius (r) of its base.

(5) $$\theta =\tan^{-1}\left(\frac{h}{r}\right)$$

### 2.5. Evaluation of Solubility and Dissolution Rate

#### 2.5.1. Evaluation of Solubility in Water

An excess amount of each sample was placed into a vial, including 10 mL of water. The vials were then agitated at room temperature (~22 °C) by a magnetic stirrer set at 500 rpm. After 24 h, the samples were passed through 0.45 μm membrane filters (PTFE Syringe Filters, Membrane-solutions, Plano, TX, USA) and diluted with 90% methanol. The amount of soluble APR was determined using high-performance liquid chromatography (HPLC), as described in Section 2.6.

#### 2.5.2. Drug Dissolution Study

An in vitro drug dissolution study was performed according to USP Dissolution Test 2. The dissolution experiments were carried out using a dissolution tester (DST-810, LABFINE, Inc., Anyang, Korea). The dissolution medium (300 mL; 0.3% SLS, sodium lauryl sulfate) was maintained at a temperature of 37 ± 0.5 °C and stirred with a paddle at a rotation speed of 50 rpm. At predetermined time intervals (0.5, 1, 2, 4, 8, and 12 h), aliquots of 1 mL were withdrawn from the flask, passed through 0.45 μm membrane filters, and immediately analyzed by HPLC, as described in Section 2.6.

### 2.6. HPLC Analysis

Concentrations of APR were determined using a Waters HPLC with the Breeze 2 analysis program (Waters, Milford, MA, USA) and a CapCell Pak C_18_ MG column (5 µm, 4.6 mm× 250 mm Shiseido, Tokyo, Japan) at 30 °C. APR was detected by its absorbance at 220 nm using a UV detector. The mobile phase was prepared by mixing methanol and water at a ratio of 90:10 (v/v), passing through a 0.45 μm nylon membrane filter, and then degassing in a sonicator for 10 min. The mobile phase was used at a flow rate of 1.0 mL/min. The injection volume was 10 µl.

### 2.7. Statistical Analysis

Three independent experiments were conducted for all analyses. The presented data (means ± standard deviations) were compared by one-way analysis of variance and Student’s *t*-tests. A value of *p* < 0.05 was considered statistically significant.

## 3. Results and Discussion

### 3.1. Instrument Characterization of PC-Based Dispersion of APR

#### 3.1.1. Fourier Transform Infrared Spectroscopy

**Figure 1:**
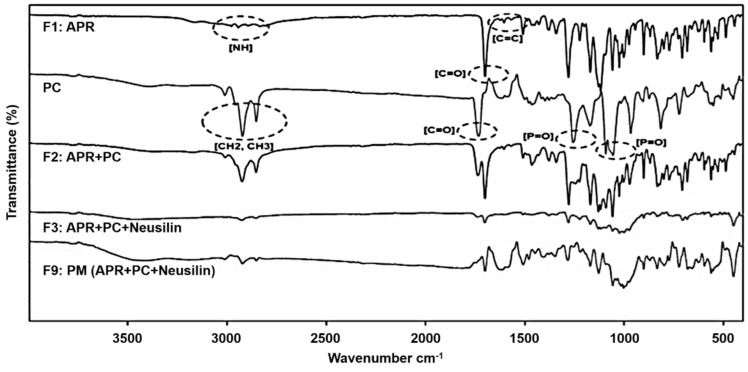
Fourier transform infrared spectroscopy (FTIR) overlay spectra of solid dispersions. F1: pure aprepitant (APR); F2: solid dispersion consisting of APR and phosphatidylcholine (PC); F3: solid dispersion consisting of APR, PC, and Neusilin^®^; F9: physical mixture (PM) of APR, PC, and Neusilin^®^; PC: solid dispersion consisting of PC.

The FTIR study was carried out to determine the chemical interactions between APR and the dispersion ingredients. Figure 1 shows the FTIR spectra of F2, F3, and F9 in comparison with F1 (pure APR) and PC. The FTIR spectrum of pure APR showed characteristic peaks at 2943, 2893, and 2834 cm^−1^ (N–H stretching); 1700 cm^−1^ (C=O stretching); and 1605 cm^−1^ (C=C stretching) as summarized in Table 2. The FTIR spectrum of F2 indicated that there were slight changes in the fingerprint region, i.e., the absorption peaks of N–H stretching to 3009, 2924, and 2853 cm^−1^; the C=O stretching remained; and the C=C stretching peak was no longer detected. The FTIR spectrum of F3 showed that the N–H stretching peaks shifted to 2925 and 2854 cm^−1^; the C=O stretching peak was shifted to 1702 cm^−1^, and the C=C stretching peak was absent. In the case of F9, the peaks for both N–H stretching and C=C stretching were not detected, but that of C=O stretching remained at 1700 cm^−1^. The difference in composition between F2 and F3 is the presence of Neusilin^®^. This suggests that the carbonyl group in APR forms an H-bond (hydrogen bond) with Neusilin^®^ but not with PC. Neusilin^®^ consists of amorphous microporous granules of magnesium aluminometasilicate and is known to be a potential compound for forming H-bonds [42]. Indeed, similar changes in FTIR spectra were previously reported by Vojinović et al. between the amide group of carbamazepine and the carbonyl group of Kollidon^®^ VA64 and/or the silanol group of Neusilin^®^ in polymer-based solid dispersions [43].

Figure 1 also shows that the FTIR spectrum of PC exhibited characteristic peaks at 1257, 1092, and 1055 cm^−1^ (P=O stretching); 1733 cm^−1^ (C=O stretching as part of the ester in the PC structure); and 2922 and 2822 cm^−1^ (CH_2_, CH_3_ stretching in the fatty acid part of the PC structure). The FTIR spectra of F2 and F3 demonstrated that the peaks for both formulations shifted to 1091 and 1058 cm^−1^ (P=O stretching); were not detected for CH_2_, CH_3_ stretching, and shifted to 1736 and 1739 cm^−1^ (C=O ester stretching), respectively. Most notably, the absorption peaks of F9 at 1057 cm^−1^ (P=O stretching) were slightly changed but different from those of F2 and F3; the peak of C=O ester stretching was absent, and the peaks of CH_2_, CH_3_ stretching were the same as those of PC. There are two possible sites in PC that can form an H-bond with an NH (secondary amine) in APR (Table 2), namely C=O (ester) and P=O (Table 3). The shifted peaks of C=O (ester) of PC for F2 and F3 were caused by forming an H-bond between the C=O (ester) of PC and an NH of APR. The formation of the H-bond between the C=O (ester) of PC and NH of APR was also confirmed by the shifted NH peaks of APR for both F2 and F3, as shown in Table 2. However, considering that the peak of C=O (ester) was slightly changed, unlike that of NH, the H-bond between the C=O (ester) of PC and NH of APR may be weak. Regarding the P=O peak of PC, its disappearance at 1257 cm^−1^ indicated that the H-bond was formed between the P=O of PC and NH of APR because the peaks of NH were also shifted and absent. This suggests that the shifted and absent peak of P=O, unlike C=O (ester), reflects a substantial interaction between the P=O of PC and an NH of APR.

The interaction between APR and PC through van der Waals forces can also be observed from the FTIR spectra. The possible sites involving van der Waals forces in PC are the CH_2_, CH_3_ groups of the fatty acids, and those in APR are the C=C of the aromatic rings. The peaks of CH_2_, CH_3_ of PC, and C=C of APR disappeared in the PC-based solid dispersions, except for the formulations of pure APR (F1) and PC as shown in Figure 1 and Table 3. Hence, APR and PC interact van der Waals forces when they become a solid dispersion.

**Figure 2:**
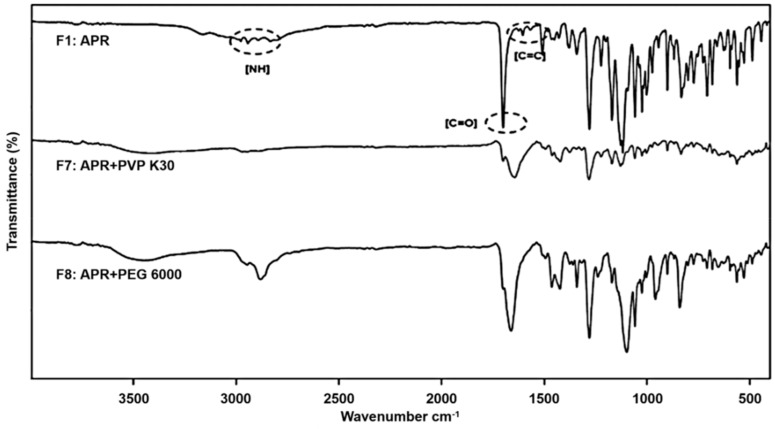
FTIR overlay spectra of solid dispersions. F1: pure aprepitant (APR); F7: solid dispersion consisting of APR and polyvinyl pyrrolidone (PVP) K30; F8: solid dispersion consisting of APR and polyethylene glycol (PEG) 6000.

In the FTIR study of traditional solid dispersions, the chemical interactions between APR and the ingredients of the polymer-based dispersions were determined (Figure 2). Table 4 shows the FTIR peaks of F7, F8, and APR. In comparison to that of pure APR, the FTIR spectrum of F7 revealed the N-H stretching peaks shifted to a single peak at 2972 cm^−1^; the C=O stretching peak at 1700 cm^−1^ remained the same; and the loss of the C=C stretching peak. For F8, the absorption peaks shifted to 2946 and 2882 cm^−1^ (N-H stretching); and 1662 cm^−1^ (C=O stretching), but the C=C stretching peak disappeared. Regarding F7, the C=O in PVP K30 formed an H-bond with the amide (NH) but not the C=O in APR as shown in Figure 2. This suggests that the amide (NH) peak of the triazolinone structure in APR only shifted among the potential H-bond sites. The APR peaks for F8, the PEG 6000-based solid dispersion, demonstrated that there were two possible H-bond sites between APR and the hydroxyl group (-OH) in PEG 6000. One of those sites was NH, and the other was C=O because both NH and C=O peaks in APR shifted, unlike those for PVP K30. The infrared studies reported by Li et al. confirmed that the H-bond forces between PEG and indomethacin were stronger than those between PVP K30 and indomethacin because PVP K30 has only one H-bonding acceptor, whereas PEG has four acceptors for H-bonds [44].

#### 3.1.2. Differential Scanning Calorimetry

**Figure 3:**
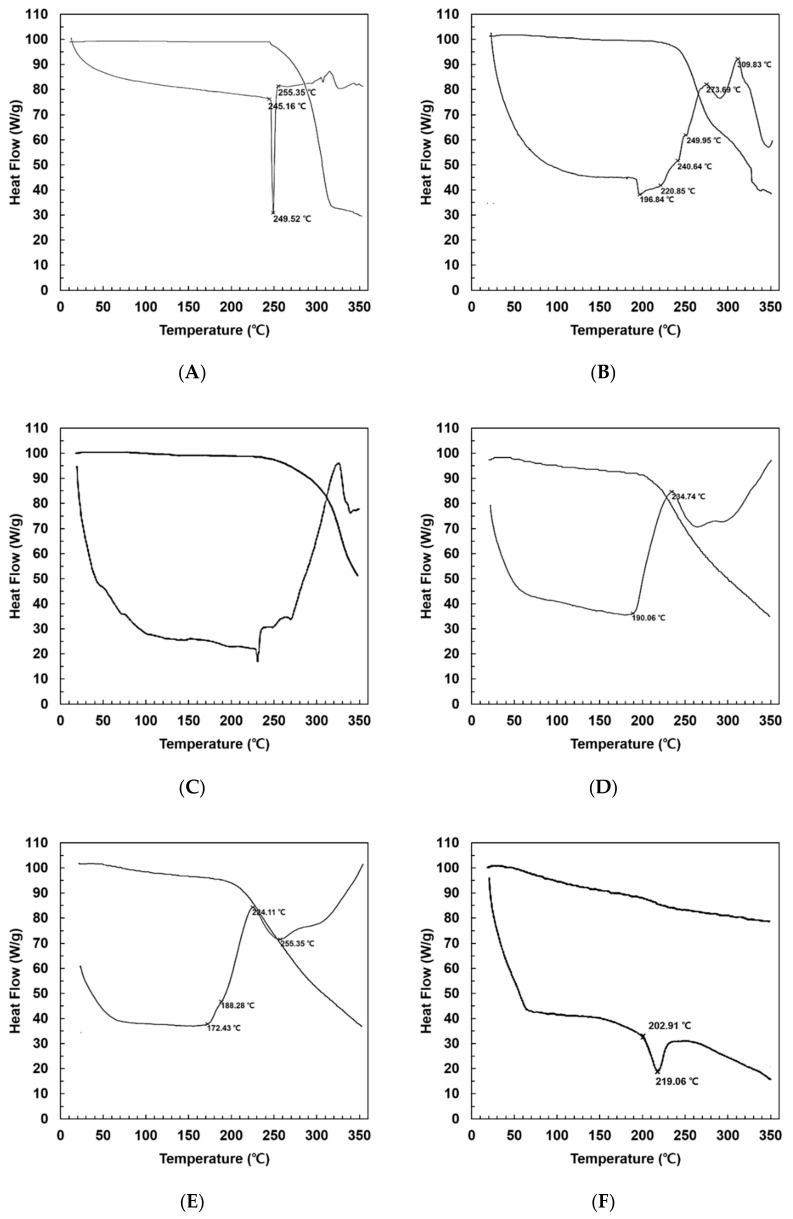
Differential scanning calorimetry (DSC) thermal analysis of aprepitant (APR)-loaded solid dispersions. Curves show the crystallinity of solid dispersion vs. physical mixture. (**A**) F1: pure APR; (**B**) F2: solid dispersion consisting of APR and phosphatidylcholine (PC); (**C**) solid dispersion consisting of PC; (**D**) F3: solid dispersion consisting of APR, PC, and Neusilin^®^; (**E**) F9: physical mixture of APR, PC, and Neusilin^®^; (**F**) pure Neusilin^®^.

DSC measurements were performed to study the physical state of APR and the ingredients of PC-based dispersions. Figure 3 shows thermograms of F2, F3, and F9 in comparison with those of F1 (pure APR), pure PC, and pure Neusilin^®^. The DSC curves for APR and Neusilin^®^ had a single endothermal peak for the melting of APR (249.5 °C) and Neusilin^®^ (219.0 °C), as shown in Figure 3A,F. PC had more than three endothermal peaks over 200 °C (Figure 3C). Regarding solid dispersions, F2 had four endothermal peaks at 196.8, 220.8, 240.6, and 249.9 °C (Figure 3B). However, F3 did not show any peaks but demonstrated an endothermal aspect from 190.6 °C to 234.7 °C (Figure 3D). According to the DSC patterns of APR and PC, the polymorphism phases of APR and PC appear to be crystalline because they demonstrated specific melting point peaks. The DSC pattern of F2 indicated that the endothermal peaks for the melting were lower than the original peaks of APR and PC, but the change to an amorphous phase was insufficient. In contrast, the endothermal peaks of F3 demonstrated that the solid dispersions changed to an amorphous phase because specific peaks were absent. This suggests that Neusilin^®^ can change the crystalline phase of APR to an amorphous phase. In addition, the increase in amorphous degree increases enthalpy but decreases physical stability, which is related to the increase in solubility and initial dissolution rate of APR in the solid dispersion [14,45].

In the case of a physical mixture with the same composition as F3, there were two melting endothermal peaks for F9 at 172.4 °C and 188.2 °C, as shown in Figure 3E. An endothermal aspect of F9 was also observed from 188.2 °C to 224.1 °C, which was similar to that of F3, but not F1, F2, or PC. The physical mixture might be a crystalline phase because two specific peaks were observed. However, the degree of the crystalline phase with F9 was lower than that with F1 and F2 because the endothermal aspect was similar to that with F3. This suggests that the reduced crystalline phase of F9 is attributable to the effect of Neusilin^®^ for the same reasons as mentioned above for F3. Regarding the reduction in melting point to 172.4 °C for F9 compared with that for F1 and F2, this indicates that porous materials, such as Neusilin^®^, also have the ability to decrease the melting point [46].

#### 3.1.3. Morphological Evaluation

**Figure 4:**
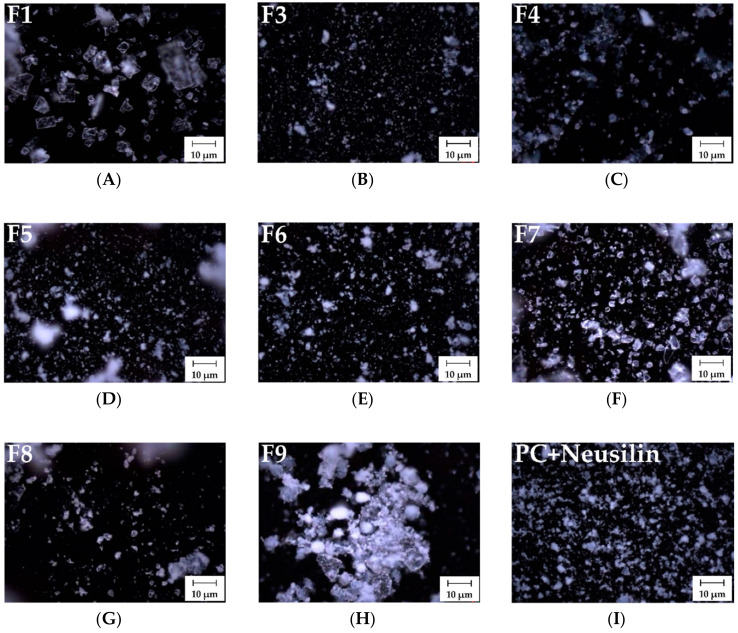
The morphology of different solid dispersions observed as surface images by microscopy. (**A**) F1: pure aprepitant (APR); (**B**) F3: solid dispersion consisting of APR, phosphatidylcholine (PC), and Neusilin^®^; (**C**) F4: solid dispersion consisting of APR, PC, and Syloid^®^; (**D**) F5: solid dispersion consisting of APR, PC, Neusilin^®^, and croscarmellose sodium; (**E**) F6: solid dispersion consisting of APR, PC, Neusilin^®^, and Kollidon^®^ CL; (**F**) F7: solid dispersion consisting of APR and PVP K30; (**G**) F8: solid dispersion consisting of APR and PEG 6000; (**H**) F9: physical mixture of APR, PC, and Neusilin^®^; (**I**) solid dispersion consisting of PC and Neusilin^®^.

Microscopy was used to compare the morphology and particle size of each formulation. Figure 4 shows the surface images by cross-polarized light of F3–F9 in comparison to those of F1 (pure APR) and the solid dispersion made by PC and Neusilin^®^. The morphology of pure APR indicated a broad plate shape of crystalline structures (Figure 4A). For F3 and F4, crystalline structures were absent, and only small particles were detected (Figure 4B,C). The morphologies of F5 and F6 revealed no crystalline particles, and the particle sizes were similar to those of F3 (Figure 4D,E). Regarding the polymer-based solid dispersions, the morphologies of F7 and F8 were crystalline structures but not of the form noted for APR (Figure 4F,G). For the physical mixture, the particle morphology of F9 was heterogeneous in form, and some of the particles looked similar to the pure APR shapes (Figure 4H). These observations are consistent with the conversion of APR crystalline phases to amorphous forms by dispersing the APR into the solid matrix. Adsorbents can prevent aggregation because a drug can access their intra-particle pores [47]. Therefore, the formulations using adsorbents produced small particles and crystal particles were not present. Regarding the effect of disintegrants, both disintegrants more homogeneously separated APR into the matrix of PC-based dispersions than formulations using only PC and adsorbents. It has been suggested that the crosslinked microfibril that facilitates water swelling creates more amorphous particles in PC-based dispersions [48]. In the polymer-based dispersion, the crystalline phase of APR decreased, but its conversion to an amorphous phase was likely insufficient. The morphological studies reported by Schachter et al. confirmed that ketoprofen-dispersed, poly(ethylene oxide)-based solid dispersions produced both crystalline and amorphous regions [49]. Therefore, although the crystalline regions of a polymer-based dispersion lead to stabilization of the formulation, they cause delayed drug dissolution due to interference.

#### 3.1.4. X-ray Powder Diffraction

**Figure 5:**
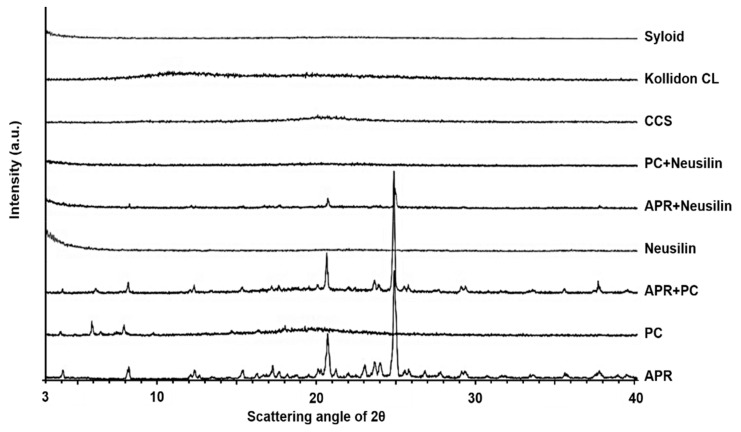
X-ray powder diffraction (XRPD) patterns of solid dispersions and ingredients. Aprepitant (APR), phosphatidylcholine (PC) as lipid carrier, Neusilin^®^, and Syloid^®^ as adsorbents, Kollidon^®^ CL, and croscarmellose sodium (CCS) as disintegrants.

**Figure 6:**
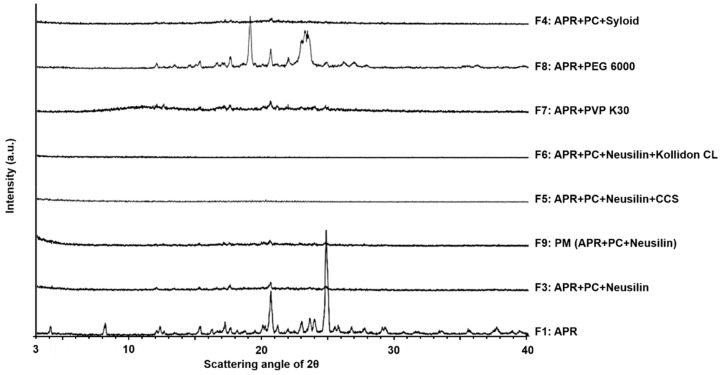
XRPD patterns of solid dispersions for the formulations from F3 to F9 and pure aprepitant (APR). Phosphatidylcholine (PC) as lipid carrier, Neusilin^®^, and Syloid^®^ as adsorbents, Kollidon^®^ CL, and croscarmellose sodium (CCS) as disintegrants.

The XRPD patterns for the pure ingredients, physical mixture, and solid dispersions of APR are shown in Figure 5 and Figure 6. The diffractogram of APR showed crystalline phases and demonstrated characteristic peaks at 8.25°, 17.22°, 20.57°, 20.65°, 22.99°, 23.62°, and 23.37° (2θ). The diffractogram of PC showed highly amorphous phases and demonstrated characteristic peaks at 3.95°, 5.93°, 6.47°, and 7.92° (2θ). In addition, the diffractograms of Neusilin^®^, Syloid^®^, CCS, and Kollidon^®^ CL as adsorbents and disintegrants demonstrated amorphous phases because there were no characteristic peaks.

The diffractogram of F2 demonstrated crystalline phases with the patterns of both APR and PC. The diffractogram of F3 showed that the crystalline phases had decreased from those of F2, revealing highly amorphous phases. Likewise, the crystallinity of F5, F6, and F9 showed amorphous phases. With respect to polymer-based solid dispersions, the diffractogram of F7 demonstrated highly amorphous phases, but not to the extent of those observed with F3. The diffractogram of F8 revealed crystalline phase patterns that were dissimilar to those of APR but similar to those of PEG 6000. Comparing the diffractograms of F2 and APR, the highly amorphous phases of PC could not sufficiently convert the crystallinity of APR to an amorphous form in the solid dispersion. PC possesses a high glass transition temperature due to its long hydrocarbon chain length, and, therefore, the crystallinity of the solid dispersion remained at body temperature [35,50]. Regarding the effect of Neusilin^®^, the diffractograms of F3, F5, F6, and F9 indicated that Neusilin^®^ can convert the crystallinity of APR to an amorphous phase in the solid dispersions. Especially, the diffractogram of the mixture of APR with Neusilin^®^ showed significant amorphous phases. The FTIR studies of Neusilin^®^ confirmed the formation of H-bonds with APR. In addition, the increased extent of H-bonding between APR and Neusilin^®^ results in a decrease in the degree of crystallinity of APR [51]. The change in the formulations from crystalline to amorphous phases could enhance the dissolution rate of APR from the solid dispersions because amorphous states of particles require lower energy to break up the crystal lattice than crystal structures do [14,52]. Regarding using Syloid^®^ as the alternative to Neusilin^®^, the diffractogram of F4 indicates that Syloid^®^ converted APR to a more strongly amorphous phase than Neusilin^®^ (i.e., compared to F3). This suggests that the degree of H-bonding between APR and Syloid^®^ is greater than that of Neusilin^®^. According to the FTIR studies reported by Vadher et al., the interaction of Neusilin^®^ with aceclofenac by H-bonding can convert the crystalline form of aceclofenac to an amorphous form [42]. The diffractograms of the formulations using disintegrants demonstrated that the degree of the amorphous state for F5 and F6 was higher than that for the formulations without disintegrant because the specific peaks for F5 and F6 decreased more than those for the formulations without disintegrant. It has been suggested that a disintegrant, as a highly swellable material, can enhance water uptake very quickly and make PC-based dispersions more amorphous particles [48].

### 3.2. Evaluation of Physical Properties of Powderized Solid Dispersions

#### 3.2.1. Measurement of Powder Density and Compressibility

The powder density and compressibility properties of PC-based dispersions powderized by adsorbents and/or disintegrants were evaluated by measuring their bulk density, tapped density, Carr’s index, and Hausner ratio. Table 5 shows the values of each property for F3, F4, F5, and F6. The results of Carr’s index showed that the values of the PC-based dispersions ranged from 11.395% to 14.405%. Hausner ratios of the PC-based formulations ranged from 1.129 to 1.168. The powder density and compressibility of F2 could not be evaluated due to its sticky texture. According to the criteria for the evaluation of the compressibility of powders or granules provided by the US Pharmacopeia, the flow property of the formulations was “good” [53]. Decreased values of compressibility for PC-based dispersions indicate better packing ability and improved flowability. It has been suggested that lower compressibility values are related to high amorphous morphologies. The compressibility results demonstrated that the values of formulations using adsorbents were higher than those using both adsorbents and disintegrants, that is, the amorphous state was greater for formulations using both adsorbents and disintegrants than for formulations without disintegrants. Therefore, the PC-based solid dispersions could be successfully powderized by adsorbents and disintegrants.

#### 3.2.2. Measurement of Flow Properties

The flowability of PC-based dispersions powderized by adsorbents and/or disintegrants was evaluated by measuring the angle of repose and flow rate after passing the samples through a funnel. Table 6 shows the values of each flow property for F3–F6. The results of the angle of repose demonstrated that the values for PC-based dispersions ranged from 31.54 to 34.71. The flowability of F2 could not be determined due to its sticky texture. Based on the criteria for the evaluation of the flowability of powders or granules provided by the US Pharmacopeia, the flow properties of the tested formulations were “good” [53]. The results of the flow rates demonstrated that all formulations ranged from 4.03–6.47 g/s, which also indicates that the formulations were able to pass through the orifice of the funnel easily. The values of the angle of repose were higher for the formulations using adsorbents than for those using both adsorbents and disintegrants, that is, the degree of stickiness of the formulations without disintegrants was higher. Consistent with these results, the flow rates of the formulations using adsorbents were lower than those of the formulations using both adsorbents and disintegrants. Comparing the formulations using adsorbents, the flow rate of F3 was higher than that of F4. This suggests that Neusilin^®^ adsorbed the formulations in its porous structure to a greater extent than Syloid^®^ did. Indeed, similar results were previously reported by Mura et al., where Neusilin^®^ US2 exhibited the highest apparent density values as well as the best flow properties. However, in the same study, Syloid^®^ 244 FP demonstrated low apparent density values and poor flow properties that were considered unacceptable [54]. Therefore, this suggests that the PC-based solid dispersions could be successfully powderized by adsorbents and disintegrants, exhibiting suitable flow properties to be formulated into solid dosage forms.

### 3.3. Evaluation of Solubility and Dissolution Rate

#### 3.3.1. Evaluation of Solubility in Water

**Figure 7:**
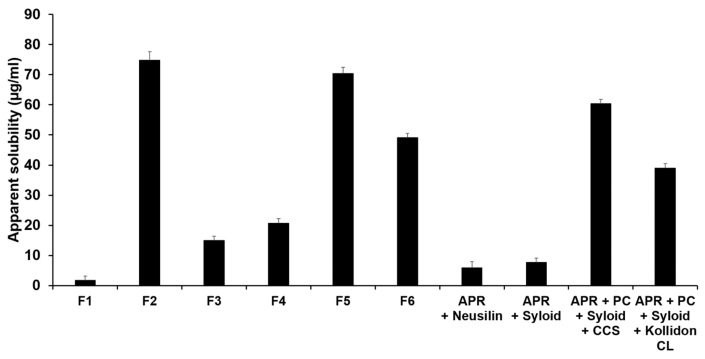
Water solubility of aprepitant (APR) in the formulations. Results are expressed as the means ± SD of three independent experiments (*n* = 3). F1: pure APR; F2: solid dispersion consisting of APR and phosphatidylcholine (PC); F3: solid dispersion consisting of APR, PC, and Neusilin^®^; F4: solid dispersion consisting of APR, PC, and Syloid^®^; F5: solid dispersion consisting of APR, PC, Neusilin^®^, and croscarmellose sodium (CCS); F6: solid dispersion consisting of APR, PC, Neusilin^®^, and Kollidon^®^ CL.

The water solubility of APR in the solid dispersions was analyzed using an HPLC method. Figure 7 shows the results of the water solubility of formulations F1 to F8. F2 had the greatest solubility in water, approximately 42 times greater than that of pure APR. The water solubility of F5 was the second-highest at approximately 40 times that of pure APR. The water solubility of F3 was noticeably low. The order of water solubility for all formulation was F2 > F5 > (APR + PC + Syloid^®^ + CCS) > F6 > (APR + PC + Syloid^®^ + Kollidon^®^ CL) > F4 > F3 > (APR + Syloid^®^) > (APR + Neusilin^®^) > F1. F3 and F4 showed that better APR solubility compared to the formulations devoid of PC (i.e., APR loaded in Neusilin^®^ or Syloid^®^) due largely to the solubilization effect by PC. The high solubility of F2 might be due to the choline phosphate in PC and the triazolinone in APR. Choline phosphate is a hydrophilic structure that can form ionic bonds with water, which leads to the easy dissolution of the PC in water with subsequent release of APR from the PC-based solid dispersion. The triazolinone in APR can also form H-bonds with PC, as shown in the FTIR studies, which enhances the solubility of APR. According to the studies reported by Gu et al., the water solubility of hesperetin was higher with the formulation using PC than with the formulation using d-α-tocopheryl polyethylene glycol 1000 succinate as a polymer [55]. Comparing the formulations using adsorbents to F2, the water solubility of F3 and F4 was lower than that of F2. Neusilin^®^ and Syloid^®^ can absorb the formulations in their porous structures, decreasing the water solubility of APR in solid dispersions. Therefore, PC-based dispersions using adsorbents might show increased stability from atmospheric moisture when they are stored. Comparing the solubility effects of Neusilin^®^ with Syloid^®^, the water solubility of F3 was lower than that of F4. According to the specifications of silica particles from Fuji Chemical Industry Co., Ltd. (Toyama, Japan) and Grace Co., Ltd., (Columbia, MD, USA) the specific surface area and pore diameter of Syloid^®^ were higher (380 m^2^/g and 17 nm, respectively) than those of Neusilin^®^ (300 m^2^/g and 5–6 nm, respectively). In addition, the particle size of Syloid^®^ was lower (3 µm) than that of Neusilin^®^ (44–177 µm) [56,57]. This suggests that the wide specific surface area and pore size of silica particles increase its wettability, thus increasing the solubilized amount of APR from the formulation. The study of powder wettability reported by Mura et al. confirmed that the wettability for the formulation made by Neusilin^®^ US2 was lower than that for the formulation made by Syloid^®^ 244 FP [54]. However, comparing the solubility of the formulations using adsorbent and disintegrant, the water solubility of F5 and F6 was higher than that of the formulations using Syloid^®^ and CCS or Kollidon^®^ CL. This suggests that the water solubility of the PC-based solid dispersion using Neusilin^®^ was lower than that using Syloid^®^, but the water solubility of the disintegrant-added formulations showed opposite results [58]. The results of the powderized self-microemulsifying drug delivery system (SMEDDS) using adsorbents and disintegrants reported by Seljak et al. confirmed that the solubility and dissolution values of SMEDDS using Neusilin^®^ US2 were higher than those of SMEDDS using Syloid^®^ 244 FP [59]. The water solubility of F5 and F6 was higher than that of F3. Disintegrants have the ability to absorb water and increase the probability of APR interacting with water. Therefore, the formulations using disintegrants had higher water solubility. In this regard, the dissolution rate of F5 might be higher than that of F3. Therefore, stronger chemical interactions of the carrier with both APR and water lead to higher water solubility of APR in the solid dispersions.

#### 3.3.2. Drug Dissolution Studies

**Figure 8:**
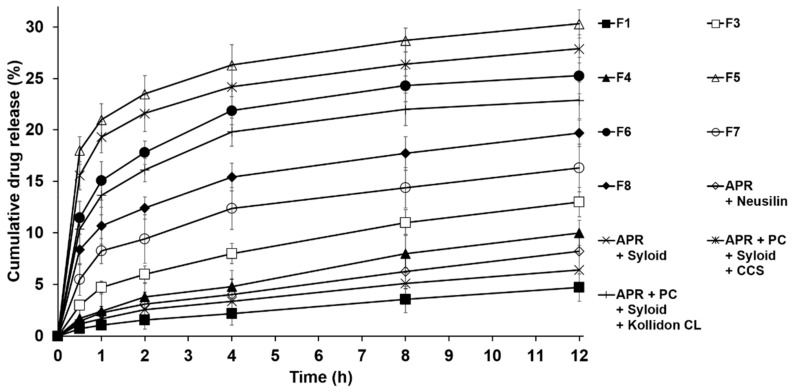
Dissolution profiles of aprepitant (APR) from solid dispersions using phosphatidylcholine (PC) in comparison with PEG 6000- or PVP K30-based solid dispersions. Results are expressed as the means ± SD of three independent experiments (*n* = 3). F1: pure APR; F3: solid dispersion consisting of APR, PC, and Neusilin^®^; F4: solid dispersion consisting of APR, PC, and Syloid^®^; F5: solid dispersion consisting of APR, PC, Neusilin^®^, and croscarmellose sodium (CCS); F6: solid dispersion consisting of APR, PC, Neusilin^®^, and Kollidon^®^ CL; F7: solid dispersion consisting of APR and PVP K30; F8: solid dispersion consisting of APR and PEG 6000.

The drug dissolution rate profiles of APR for the solid dispersions in dissolution media (0.3% SLS) were obtained as outlined in USP Dissolution Test 2. Figure 8 shows the drug dissolution results of the formulations F1 to F8. The dissolution rates for all formulations were higher than that for pure APR in dissolution media. The APR dissolution rate of F5 was the greatest among the tested formulations and was approximately 6.5 times higher than that of pure APR. When compared to the dissolution rate of F3, the rate of F5 was ~2 times higher. This characteristic could be attributed to the high hygroscopicity of CCS as the hydrophilic ingredient. CCS quickly absorbs water into its microfibrils and acts as a hydrophilic channel drawing liquid into the solid dispersion by swelling, thus accelerating dissolution of APR [60,61]. With the formulations using disintegrants, the dissolution rate of APR from the solid dispersion using CCS was significantly higher than that of the formulation using Kollidon^®^ CL. This suggests that the hydrophilic property of CCS is higher than that of Kollidon^®^ CL [62]. In this sense, the hydroxyl group (-OH) of CCS might interact more strongly with water than the C=O of Kollidon^®^ CL [63]. The drug release rate was higher for F3 than for F4. Additionally, the drug release rates were higher for F5 and F6 than for the formulations using (Syloid^®^ and CCS) and (Syloid^®^ and Kollidon^®^ CL), respectively. This suggests that Syloid^®^ keeps APR in its pores more strongly than Neusilin^®^, as mentioned above in the XRPD study. Therefore, the dissolution rate of APR from the formulation using Neusilin^®^ was higher than that from the formulation using Syloid^®^. Indeed, similar results were previously reported by Alwadei et al. [58] and Seljak et al. [59] with self-micro/nanoemulsifying drug delivery systems, where the drug release rates of the formulations using Neusilin^®^ US2 were higher than those of the formulations using Syloid^®^ 244 FP, where formulations included disintegrants. When comparing APR dissolution rates of F3 and F4 (PC containing APR dispersions in Neusilin^®^ or Syloid^®^) with APR dispersions in Neusilin^®^ or Syloid^®^, without PC, the dissolution rates were higher for F3 and F4 than for the formulations composed of APR and Neusilin^®^ or Syloid^®^. This clearly suggests that PC can act as solubility enhancer due to its amphiphilic nature and the increased solubilization effect of APR resulted in the faster APR release. Regarding polymer-based solid dispersions, the drug release rates of F7 and F8 were significantly higher than those of F1, F3, and F4 but not to the extent of the formulation using PC, Syloid^®^, and Kollidon^®^ CL. This suggests that the hydrophilic ingredients, such as PVP K30 and PEG 6000, can facilitate absorption of water into the microfibrils and, therefore, the dissolution rate of APR from the solid dispersion was improved. Comparing both polymer-based solid dispersions, the drug release rate of F8 was significantly higher than that of F7. This suggests that the hydrophilic property of PEG 6000 is better than that of PVP K30. This is indicative of the hydroxyl groups (-OH) in PEG 6000 interacting with both the NH (secondary amine) and C=O in APR, while the C=O groups in PVP K30 form H-bonds with only the NH (secondary amine) in APR, as shown in the FTIR studies. Thus, the APR-loaded and PC-based solid dispersions prepared with Neusilin^®^ and CCS as adsorbents and disintegrants, respectively, are expected to exhibit superior solubility and dissolution rates for APR in water.

## 4. Conclusions

In the present research, an attempt was made to improve the solubility and dissolution of APR, a poorly water-soluble drug. For this purpose, solid dispersions were prepared by using PC, a phospholipid, as the carrier, and a solvent evaporation technique. To improve the physical properties of the solid dispersion, Neusilin^®^ and CCS were utilized as the adsorbent and disintegrant, respectively. The results of FTIR confirmed that APR interacted with PC by both H-bonds and van der Waals forces that can also be seen between APR and the polymer carriers, PEG 6000 and PVP K30. Morphology, DSC, and XRPD analyses verified that the use of PC is not sufficient to convert the crystallinity of APR to an amorphous phase, while the formulations with adsorbent and disintegrant were converted to amorphous phases. Regarding the polymer-based solid dispersions, both PVP K30 and PEG 6000 were not able to convert the crystallinity of APR to an amorphous phase, but they were crystalline on their own. The physical property studies of the PC-based dispersions demonstrated that the formulation using PC alone was “sticky”, but the formulations using adsorbents and disintegrants were successfully powderized with good flow properties based on the US Pharmacopeia. The solubility studies confirmed that the PC-based solid dispersions could solubilize APR in water better than pure APR. The dissolution studies confirmed that the dissolution rate of APR from the PC-based solid dispersions that included both adsorbent and disintegrant was the greatest among all tested formulations. Therefore, the PC-based solid dispersions are promising delivery systems to promote the absorption and bioavailability of APR for oral administration.

## Figures and Tables

**Table 1:** The composition of solid dispersion formulations of APR.

	APR (mg)	PC (mg)	Adsorbent		Disintegrant		Polymeric Carriers		Control
			Neusilin^®^ (mg)	Syloid^®^ (mg)	CCS (mg)	Kollidon^®^ CL (mg)	PVP K30 (mg)	PEG 6000 (mg)	
F1	100								
F2	100	100							
F3	100	100	200						
F4	100	100		200					
F5	100	100	200		50				
F6	100	100	200			50			
F7	100						300		
F8	100							300	
F9	100	100	200						PM *

APR, aprepitant; PC, phosphatidylcholine; CCS, croscarmellose sodium; PVP K30, polyvinylpyrrolidone K30; PEG 6000, polyethylene glycol 6000; PM, physical mixture.

**Table 2:** Infrared absorption wavenumbers (cm^−1^) of NH; C=C; and C=O stretching in APR and different formulations.

	NH (cm^−1^)	C=C (cm^−1^)	C=O (cm^−1^)
APR	2943, 2893, 2834	1605	1700
F2	3009, 2924, 2853	-	1700
F3	2925, 2854	-	1702
F9	-	-	1700

**Table 3:** Infrared absorption wavenumbers (cm^−1^) of CH_2_, CH_3_; C=O; and P=O stretching in PC and different formulations.

	CH_2_, CH_3_ (cm^−1^)	C=O (ester) (cm^−1^)	P=O (cm^−1^)
PC	2922, 2822	1733	1257, 1092, 1055
F2	-	1736	1091, 1058
F3	-	1739	1091, 1058
F9	2922, 2822	-	1057

**Table 4:** Infrared absorption wavenumbers (cm^−1^) of NH; C=C; and C=O stretching for aprepitant (APR) in polyvinyl pyrrolidone (PVP) K30 (F7) and polyethylene glycol (PEG) 6000 (F8) solid dispersions.

	NH (cm^−1^)	C=C (cm^−1^)	C=O (cm^−1^)
APR	2943, 2893, 2834	1605	1700
F7	2972	-	1700
F8	2946, 2882	-	1662

**Table 5:** Bulk density, tapped density, Carr’s index, and Hausner ratio of solid dispersions. Results are expressed as means ± SD of three independent experiments (*n* = 3).

	Bulk Density (g/mL)	Tapped Density (g/mL)	Carr’s Index (%)	Hausner Ratio
F3	0.155 ± 0.001	0.180 ± 0.001	13.864 ± 0.096	1.161 ± 0.001
F4	0.172 ± 0.001	0.201 ± 0.001	14.405 ± 0.089	1.168 ± 0.001
F5	0.171 ± 0.004	0.193 ± 0.005	11.395 ± 0.164	1.129 ± 0.002
F6	0.166 ± 0.002	0.189 ± 0.003	12.500 ± 0.143	1.143 ± 0.002

**Table 6:** Angles of repose and flow rates of solid dispersions measured after pouring the samples into a funnel with a 10-mm diameter orifice. Results are expressed as means ± SD of three independent experiments (*n* = 3).

	Angle of Repose (θ)	Flow Rate (g/s)
F3	33.81 ± 0.43	4.57 ± 0.25
F4	34.71 ± 0.89	4.03 ± 0.32
F5	31.54 ± 0.32	6.47 ± 0.30
F6	32.17 ± 0.43	5.21 ± 0.31
